# Inflammatory Regulation by Driving Microglial M2 Polarization: Neuroprotective Effects of Cannabinoid Receptor-2 Activation in Intracerebral Hemorrhage

**DOI:** 10.3389/fimmu.2017.00112

**Published:** 2017-02-14

**Authors:** Li Lin, Tao Yihao, Feng Zhou, Niu Yin, Tan Qiang, Zheng Haowen, Chen Qianwei, Tang Jun, Zhang Yuan, Zhu Gang, Feng Hua, Yang Yunfeng, Chen Zhi

**Affiliations:** ^1^Department of Neurosurgery, Nanchong Central Hospital, Nanchong, China; ^2^Department of Neurosurgery, Southwest Hospital, Third Military Medical University, Chongqing, China; ^3^Department of Neurosurgery, Southwest Medical University Affiliated Hospital, Southwest Medical University, Luzhou, China; ^4^Department of Neurosurgery, Sichuan Provincial Corps Hospital, Chinese People’s Armed Police Forces, Leshan, China

**Keywords:** intracerebral hemorrhage, cannabinoid receptor-2, neuroinflammation, microglial polarization, CREB

## Abstract

The cannabinoid receptor-2 (CB2R) was initially thought to be the “peripheral cannabinoid receptor.” Recent studies, however, have documented CB2R expression in the brain in both glial and neuronal cells, and increasing evidence suggests an important role for CB2R in the central nervous system inflammatory response. Intracerebral hemorrhage (ICH), which occurs when a diseased cerebral vessel ruptures, accounts for 10–15% of all strokes. Although surgical techniques have significantly advanced in the past two decades, ICH continues to have a high mortality rate. The aim of this study was to investigate the therapeutic effects of CB2R stimulation in acute phase after experimental ICH in rats and its related mechanisms. Data showed that stimulation of CB2R using a selective agonist, JWH133, ameliorated brain edema, brain damage, and neuron death and improved neurobehavioral outcomes in acute phase after ICH. The neuroprotective effects were prevented by SR144528, a selective CB2R inhibitor. Additionally, JWH133 suppressed neuroinflammation and upregulated the expression of microglial M2-associated marker in both gene and protein level. Furthermore, the expression of phosphorylated cAMP-dependent protein kinase (pPKA) and its downstream effector, cAMP-response element binding protein (CREB), were facilitated. Knockdown of CREB significantly inversed the increase of M2 polarization in microglia, indicating that the JWH133-mediated anti-inflammatory effects are closely associated with PKA/CREB signaling pathway. These findings demonstrated that CB2R stimulation significantly protected the brain damage and suppressed neuroinflammation by promoting the acquisition of microglial M2 phenotype in acute stage after ICH. Taken together, this study provided mechanism insight into neuroprotective effects by CB2R stimulation after ICH.

## Introduction

Intracerebral hemorrhage (ICH) is a subtype of stroke with high morbidity and mortality, accounting for about 15% of all deaths from strokes (1). Pronounced inflammatory reactions play an important role in secondary brain injury following ICH (2, 3); various stimuli, including thrombin or glutamate, activate microglia and initiate an inflammatory response, and subsequently release pro-inflammatory cytokines or chemokines to enhance neuroinflammation (3, 4).

Microglia are the resident macrophages of the brain and the first responders of the immune system (5). They are highly plastic cells that can assume diverse phenotypes and engage different functional programs in response to specific microenvironmental signals. In particular, stimulation with interferon-γ promotes classically activated microglia/macrophage (M1 phenotype) that release destructive pro-inflammatory mediators, such as tumor necrosis factor-α (TNF-α) and interleukin-1β (IL-1β), causing damage to healthy cells and tissues (6). In contrast, cytokines such as interleukin-4 and interleukin-10 (IL-10) induce an alternative activated microglia (M2 phenotype) that generates anti-inflammatory cytokines, such as transforming growth factor beta (TGF-β) and IL-10, which possess neuroprotective properties (7, 8). Microglia activation and M1/M2 polarization have been reported in several types of acute central nervous system (CNS) injury, such as traumatic brain injury, spinal cord injury, and ischemic stroke (9, 10). Microglia activation and polarization also occur in hemorrhagic stroke (11, 12); however, their underlying mechanisms after ICH remain unclear.

The cannabinoid receptor-2 (CB2R) signaling pathway plays an important role in CNS injury via regulation of microglial activities (13, 14). In addition, the expression of CB2R on microglia mainly depends on the activation state of microglia (15). CB2R on activated microglia have been shown to modulate properties of microglial migration and infiltration into the CNS during active neuroinflammation and degeneration (16). In our previous study, we showed that CB2R stimulation attenuated microglial accumulation after germinal matrix hemorrhage (GMH) in neonatal rats (17). However, whether CB2R has effects on microglia polarization following ICH remains unknown.

In this study, we investigated the therapeutic effects of CB2R stimulation in acute phase following experimental ICH and its related mechanisms. We found that selective stimulation of CB2R significantly protected brain injury and suppressed neuroinflammation by promoting M2 polarization of microglia. In addition, we also demonstrated that PKA/CREB signaling pathway was involved in, at least partially, the suppression of neuroinflammation. These results indicated that CB2R may be a potential therapeutic target for ICH.

## Animals and Methods

### Animals

Excluding 6 rats that died of an overdose of anesthetic before modeling, total 213 adult male Sprague-Dawley rats (250–300 g) were used for this study. Rats were housed under specific pathogen-free conditions and had free access to food and water. Animals were sacrificed at the endpoint under deep anesthesia using an overdose of intraperitoneal pentobarbital. All efforts were made to minimize suffering and animal numbers according to the *Guide for the Care and Use of Laboratory Animals*; the study was approved by the Animal Care and Use Committee at the Third Military Medical University.

### Experimental Design

#### Experiment I

**Figure 1:**
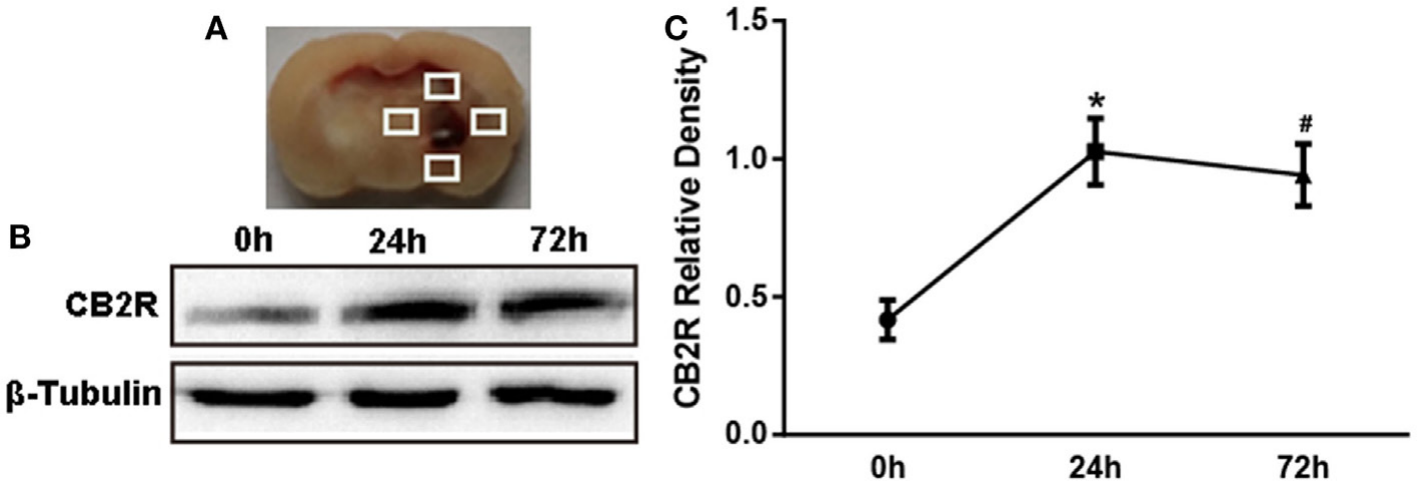
**The expression of cannabinoid receptor-2 (CB2R) after intracerebral hemorrhage (ICH)**. The coronal section of specimen **(A)**. Western blot test **(B)** of CB2R expression in total tissue around the hematoma (shown as the white quadrangles) at 0, 24, and 72 h after ICH, and the relative densitometric values analysis **(C)**. Values were expressed as the mean ± SD, *n* = 6 in per group. *P < 0.05 vs. 0 h group; ^#^*P* > 0.05 vs. 24 h group.

To determine the expression time course of CB2R after ICH, 18 rats were randomly assigned into three groups: ICH 0 h (*n* = 6), ICH 24 h (*n* = 6), and ICH 72 h (*n* = 6). Perihematomal tissue (shown as the white quadrangle in Figure 1A) was collected to detect the protein expression of CB2R.

#### Experiment II

A total of 168 rats were randomly divided into four groups for the mechanism study: sham-operated (Sham group, *n* = 42), ICH + Vehicle (ICH + Vehi group, *n* = 42), ICH + JWH133 (ICH + JWH group, *n* = 42), and ICH + JWH133 + SR144528 (ICH + JWH + SR group, *n* = 42). Sham group received only a needle insertion. The ICH + Vehi group received an equal volume of vehicle, and the ICH + JWH group received an intraperitoneal injection of a selective CB2R agonist, JWH133 (1.5 mg/kg, Tocris Bioscience, Minneapolis, MN, USA) at 1 h after surgery. The ICH + JWH + SR group was treated with a selective CB2R antagonist, SR144528 (3 mg/kg, Santa Cruz Biotechnologies, Dallas, TX, USA) 3 min before JWH133 (1.5 mg/kg) intraperitoneally. Brain water content (*n* = 6 in each group) and neurological scores (*n* = 6 in each group), including the modified Neurological Severity Score (mNSS) and forelimb placing test, were tested at 24 and 72 h after ICH. Gene levels of microglial M1/M2 markers and inflammatory cytokines in perihematomal tissue were evaluated at 6, 12, 24, and 72 h after ICH (*n* = 3 in each group). Western blots (*n* = 6 per group) and immunofluorescence staining (*n* = 6 per group) for protein expression of the tissues around the hematoma were conducted at 24 h post-ictus. The use of JWH133 and SR144528 was according to the publication (18).

#### Experiment III

For further study of the mechanism, 27 rats were randomly assigned into three groups: ICH + JWH133 + Vehicle group (JWH + Vehi, *n* = 9), ICH + JWH133 + CREB-1 siRNA group (JWH + si-CREB, *n* = 9), and ICH + JWH133 + scrambled siRNA group (JWH + scr-siRNA, *n* = 9). siRNA dilution buffer (Santa Cruz Biotechnologies, Dallas, TX, USA), CREB-1 siRNA (Santa Cruz Biotechnologies, Dallas, TX, USA), or scrambled siRNA (Santa Cruz Biotechnologies, Dallas, TX, USA) was intracerebroventricularly injected at 24 h before ICH modeling, and JWH133 was injected 1 h after surgery. PCR for gene levels (*n* = 3 in each group) and western blots for protein levels (*n* = 6 in each group) were performed at 24 h after ICH in each group.

### ICH Model

To induce ICH, rats were anesthetized with an intraperitoneal injection of 5% chloral hydrate (350 mg/kg). A feedback-controlled heating pad was used to maintain body temperature at 37.0°C. A cranial burr hole (about 1 mm) was drilled, and a 29-gauge needle was inserted stereotaxically into the right basal ganglia (coordinates: 0.2 mm anterior, 5.5 mm ventral, and 4.0 mm lateral to the bregma) (19, 20). A total of 100 µl autologous arterial blood was infused at a rate of 10 µl/min using a microinfusion pump in each rat. The sham groups received only a needle injection into the right basal ganglia.

### Brain Water Content Measurement

Brain water content was examined 24 and 72 h after surgery. As previously described (21), animals were anesthetized with an intraperitoneal injection of 5% chloral hydrate (350 mg/kg). Brains were removed, and the tissue was sliced coronally (4 mm thickness) around the hematoma. Samples were divided into four parts: ipsilateral basal ganglia, ipsilateral cortex, contralateral basal ganglia, and contralateral cortex. Cerebellum was regarded as the internal control. Sample weights were determined immediately after removal and after drying for 24 h in a 100°C oven using an electric analytical balance. Brain water content (%) was calculated as (wet weight − dry weight)/wet weight × 100%.

### Assessment of Behavioral Outcome

Behavioral outcomes were assessed in a blinded fashion at 24 and 72 h after surgery. The neurological abnormalities were assessed by the mNSS method. The evaluation was performed by an investigator blinded to the experimental scheme. The mNSS is a composite test of motor, sensory, and balance functions. Neurological function was graded on a scale of 0–18 (normal score, 0; maximal deficit score, 18) (22). For the forelimb placing test, each rat was tested 10 times for each forelimb; the percentage of trials that the rat placed the appropriate forelimb at the edge of the countertop in response to the vibrissae stimulation was determined. Testers were experienced and blind to the condition of the animal. The mean neurological score was evaluated by two blinded observers (23).

### Fluoro-Jade C and Terminal dUDP Nick End Labeling (TUNEL) Staining and Cell Counting

Fluoro-Jade C staining was used to assess neuronal degeneration. Briefly, sections were rinsed for 5 min in basic alcohol, followed by a 2-min rinse in 70% alcohol. Then, the sections were rinsed in distilled water and incubated in 0.06% KMnO_4_ for 10 min. They were rinsed in distilled water to remove excess KMnO_4_ and incubated in 0.0001% Fluoro-Jade C stain (Millipore, Boston, MA, USA) stain in 0.1% acetic acid for 10 min. Following Fluoro-Jade C labeling, the sections were rinsed three times in distilled water, air dried for 10 min, cleared in xylene, and covered with DPX. TUNEL staining was performed using an *in situ* cell death detection kit-POD (Roche, Switzerland) to reveal DNA damage according to the manufacturer’s instruction (24). High-power images (×40 magnification) were taken around the hematoma using a digital camera. Fluoro-Jade C and TUNEL-positive cells were counted. Counts were performed on four areas in each brain section.

### Real-Time PCR

PCR was performed and analyzed as previously described (25). Total RNA from brain tissue around the hematoma was extracted using Qiagen RNeasy mini kits. One microgram of RNA was reverse-transcripted and cDNA was synthesized using the PrimescriptTM RT kit (Takara, Dalian, China), substituting DNase and RNase-free water for no-RT controls. qPCR reactions were set up in 25 µl using SYBR Premix Ex TaqII kit (Takara, Dalian, China) and conducted on a CFX-96 Real-Time PCR Detection System (Bio-Rad, Hercules, CA, USA). The running procedure was 30 s at 95°C, 40 cycles of 5 s at 95°C, and 30 s at 60°C, following a melt curve. The qPCR primers were listed in Table S1 in Supplementary Material. Gene expression was quantified with standard samples and normalized with GAPDH. The data are expressed as normalized messenger RNA (mRNA) expression (fold mRNA increase).

### Immunofluorescence Staining

Immunofluorescence staining was performed as previously described (21). Briefly, free-floating slices were incubated with primary goat anti-Iba1 (1:200, Abcam, Cambridge, United Kingdom) at 4°C overnight, followed by Alexa 555-labeled rabbit anti-goat IgG (H + L) (1:500; Beyotime, Wuhan, China) secondary antibody (1 h, 37°C). Sections were washed and blocked with 10% normal goat serum for 1 h, then incubated overnight with mouse anti-CD68 (1:200, 1:500, AbD Serotec, Oxford, UK) or rabbit anti-CD206 (1:400, Santa Cruz Biotechnologies, Dallas, TX, USA). Finally, sections were incubated with the appropriate secondary antibodies for 1 h at 37°C. Colocalization was examined using a fluorescent microscope (Zeiss, LSM780).

### Western Blot Analysis

Western blot assays were performed as described previously (26). A total of 50 µg of prepared protein was loaded into each lane of SDS-PAGE gels. Gel electrophoresis was performed, and protein was transferred to a nitrocellulose membrane. The membrane was blocked in Carnation^®^ non-fat milk and probed with primary and secondary antibodies. The following primary antibodies were used: rabbit anti-CB2R (1:500, Abcam, Cambridge, UK), mouse anti-CD68 (1:500, AbD Serotec, Oxford, UK), rabbit anti-CD206 (1:500, Santa Cruz Biotechnologies, Dallas, TX, USA), rabbit anti-phospho-CREB (Ser133; 1:1,000, Cell Signaling Technology, Boston, MA, USA), rabbit anti-phospho-PKAC (Thr197; 1:1,000, Cell Signaling Technology, Boston, MA, USA), rabbit anti-β-tubulin (1:1,000, Abcam, Cambridge, UK), and mouse anti-β-actin (1:1,000, Santa Cruz Biotechnologies, Dallas, TX, USA). Then, membranes were incubated in the appropriate HRP-conjugated secondary antibody (diluted 1:1,000 in secondary antibody dilution buffer) for 1 h at 37°C. Protein bands were visualized using a nickel-intensified DAB solution, and the densitometric values were analyzed using Image J software. The housekeeping proteins β-tubulin and β-actin were used as internal controls.

### Intracerebroventricular Infusion

Intracerebroventricular infusion was performed as described previously (27). Rats were anesthetized with an intraperitoneal injection of 5% chloral hydrate (350 mg/kg). The needle of a 10-µl Hamilton syringe (Microliter No. 701; Hamilton Company) was inserted through a burr hole in the skull into the left lateral ventricle, according to the following coordinates: 1.5 mm posterior, 4.2 mm ventral, and 0.8 mm lateral to the bregma. CREB-1 siRNA or an irrelevant scrambled siRNA [500 pmol each in 1 µl siRNA dilution buffer (Santa Cruz Biotechnology)] was injected (0.5 µl/min) using a microinfusion pump at 24 h before ICH induction. The needle was removed 10 min later to prevent backflow. The burr hole was sealed with bone wax, and skin incisions were closed with sutures after the needle was removed. All rats received JWH133 (1.5 mg/kg) intraperitoneally 1 h after ICH. CREB-1 siRNA consists of three different siRNA duplexes to improve the knockdown efficiency. All CREB-1 siRNA sequences were provided in 5′–3′ orientation as shown in Table S2 in Supplementary Material.

### Statistical Analysis

Data are reported as the mean ± SD. Data were analyzed using one-way analysis of variance tests followed by Student–Newman–Keuls tests. A non-parametric test (Kruskal–Wallis H) was used if the data were not normally distributed, followed by a Nemenyi test when a two-group comparison was necessary. Differences were considered statistically significant at *P* < 0.05.

## Results

### CB2R Was Upregulated after ICH Injury

We first investigated whether CB2R alterations would respond to brain injury after ICH. Data showed that CB2R levels around hematoma (Figure 1A) were significantly increased at 24 h after ICH when compared with the ICH 0 h group (*P* < 0.05) and remained at a high level until 72 h (Figures 1B,C).

### JWH133 Reduced Brain Water Content and Improved Neurobehavioral Outcomes both at 24 and 72 h after ICH

**Figure 2:**
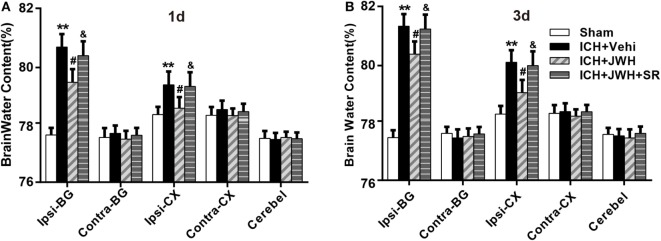
**Brain water content after intracerebral hemorrhage (ICH)**. Brain water content in ipsilateral basal ganglia at 24 h **(A)** and 72 h **(B)** after ICH. Ipsi-BG, ipsilateral basal ganglia; Ipsi-CX, ipsilateral cortex; Cont-BG, contralateral basal ganglia; and Cont-CX, contralateral cortex; Cerebel, Cerebellum; Vehi, Vehicle; JWH, JWH133; SR, SR144528. Values were expressed as the mean ± SD, *n* = 6 in each group. **P < 0.01 compared with sham group; ^#^*P* < 0.05 compared with ICH + Vehi group; ^&^*P* < 0.05 compared with ICH + JWH group.

**Figure 3:**
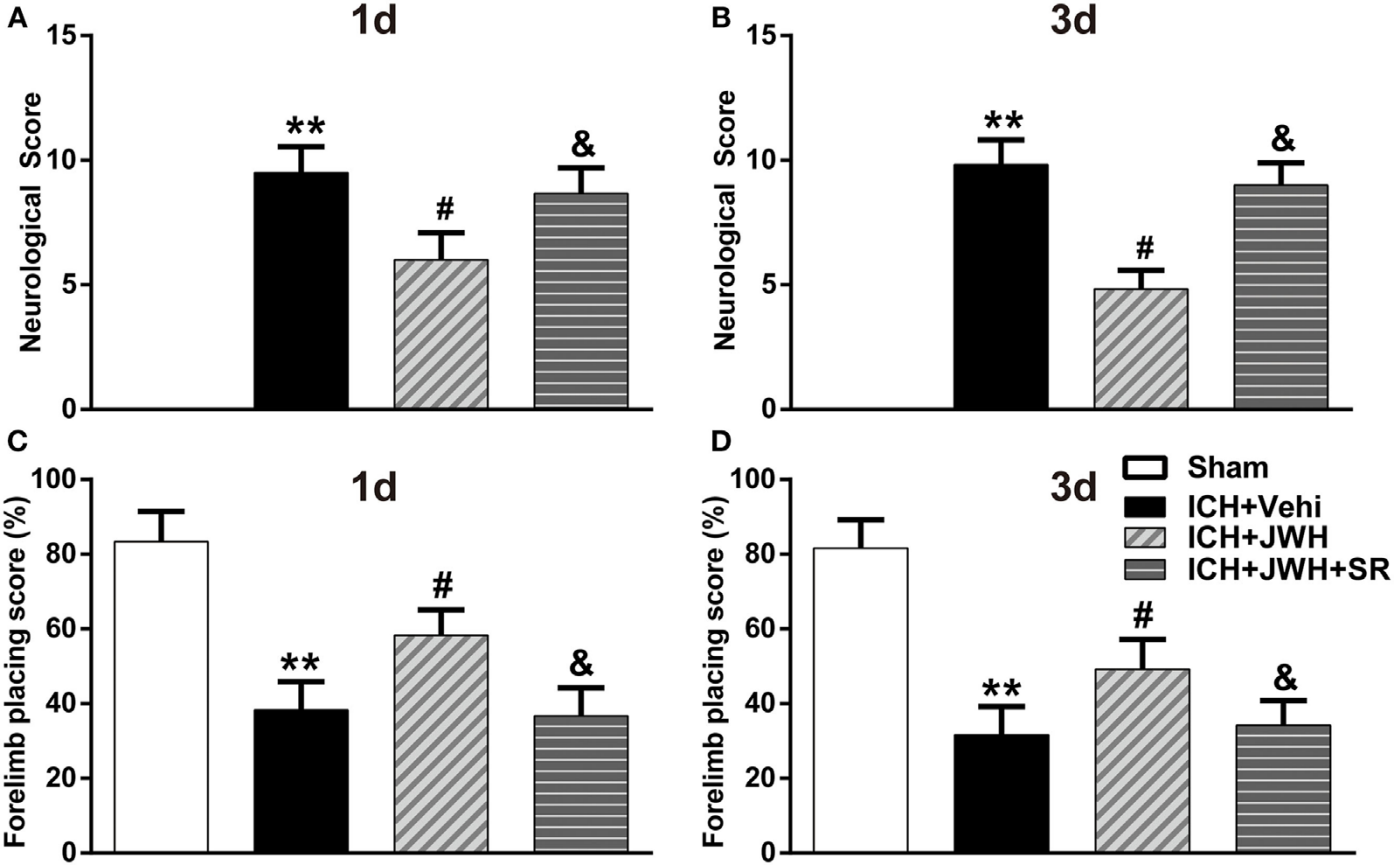
**Neurological deficit scores**. Results of modified Neurological Severity Score test at 24 h **(A)** and 72 h **(B)**, and forelimb placing test at 24 h **(C)** and 72 h **(D)**. Each experiment was repeated three times and the average value was taken for statistics. Vehi, Vehicle; JWH, JWH133; SR, SR144528. Values were expressed as the mean ± SD, *n* = 6 per group. **P < 0.01 compared with sham group; ^#^*P* < 0.05 compared with ICH + Vehi group; ^&^*P* < 0.05 compared with ICH + JWH group.

To assess brain edema and neurobehavioral outcomes after ICH, brain water content and behavioral testings, including mNSS test and forelimb placing test, were used. Data showed that both at 24 and 72 h after surgery, rats subjected to ICH had increased perihematomal edema in the ipsilateral basal ganglia (*P* < 0.01 vs. Sham; Figures 2A,B). The animals also had significantly worse mNSS test performance (*P* < 0.01 vs. Sham; Figures 3A,B) and forelimb placing scores (*P* < 0.01 vs. Sham; Figures 3C,D). In ICH + JWH groups, the perihematomal brain edemas were significantly reduced (*P* < 0.05 vs. ICH + Vehi; Figures 2A,B), and the neurobehavioral outcomes were significantly improved (*P* < 0.05 vs. ICH + vehi; Figures 3A–D). All of these JWH133 effects were reversed by SR144528.

### JWH133 Attenuated Neuronal Death and DNA Damage after ICH

**Figure 4:**
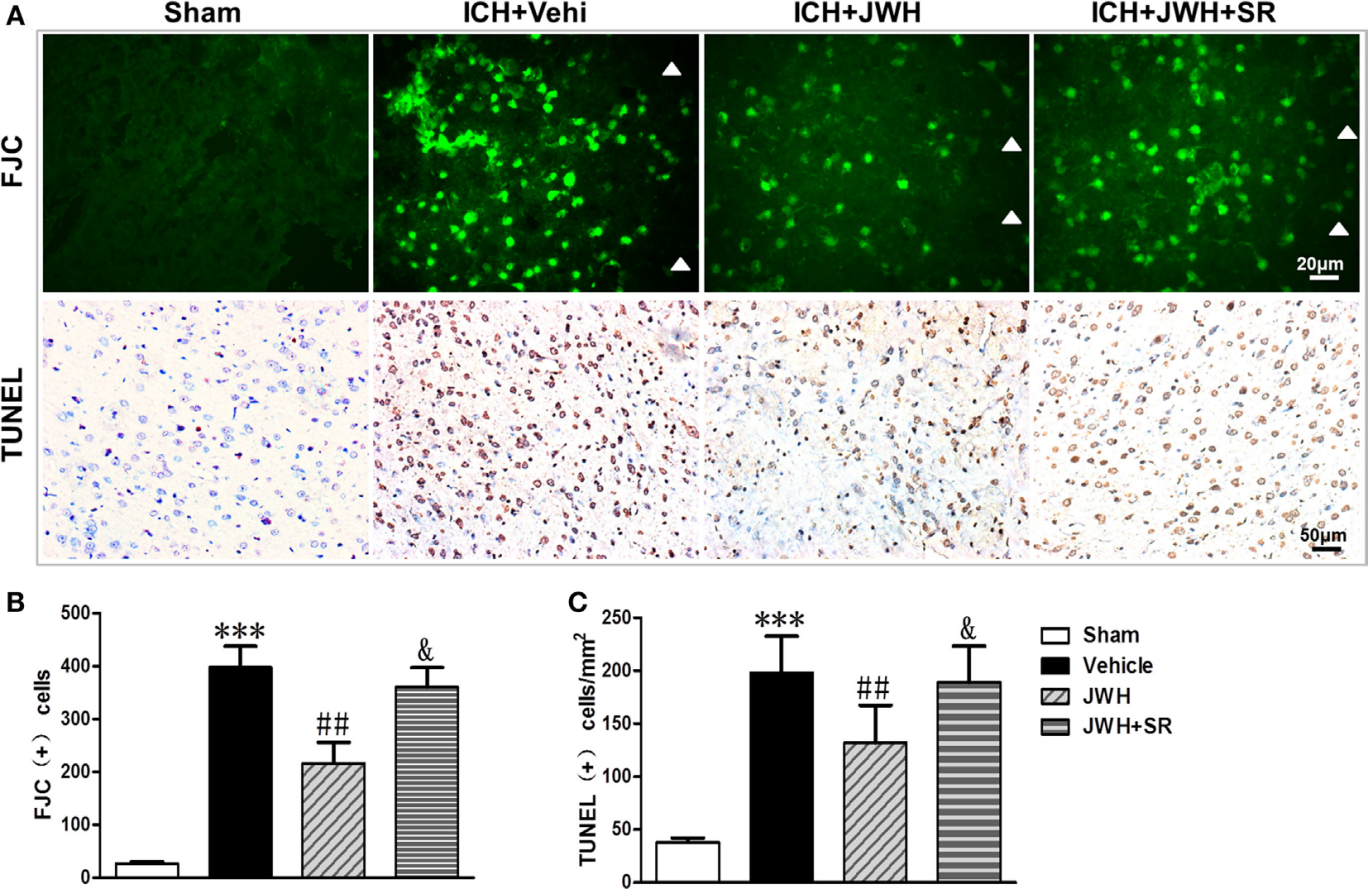
**Cell death detection following intracerebral hemorrhage (ICH)**. Fluoro-Jade C and terminal dUDP nick end labeling (TUNEL) staining for tissue around the hematoma in the ipsilateral basal ganglia at 24 h after ICH **(A)**, and analysis of positive cell counting of Fluoro-Jade C (green) **(B)** and TUNEL (brown) **(C)** Green represents for FJC-positive cells and brown represents for TUNEL-positive cells. FJC, Fluoro-Jade C; Vehi, Vehicle; JWH, JWH133; SR, SR144528. Values were expressed as mean ± SD, *n* = 6 in each group. **P < 0.01 compared with sham group; ^#^*P* < 0.05 compared with ICH + Vehi group; ^&^*P* < 0.05 compared with ICH + JWH group.

To investigate the neuronal degeneration after ICH, Fluoro-Jade C and TUNEL staining were used. At 24 h after ICH, the treatment of JWH133 significantly decreased the number of Fluoro-Jade C-positive cells (497 vs. 720/mm^2^ in ICH + Vehi group, *P* < 0.01) (Figures 4A,B). Similarly, the number of TUNEL-positive cells were significantly decreased in the ICH + JWH group (524 vs. 325/mm^2^ in ICH + Vehi group, *P* < 0.01) (Figures 4A,C). The result indicated that JWH133 could significantly attenuate neuronal death and DNA damage after ICH.

### Microglia Primarily Polarized to Classic M1 Phenotype during the ICH Acute Phase, and JWH133 Promoted M2 Polarization

**Figure 5:**
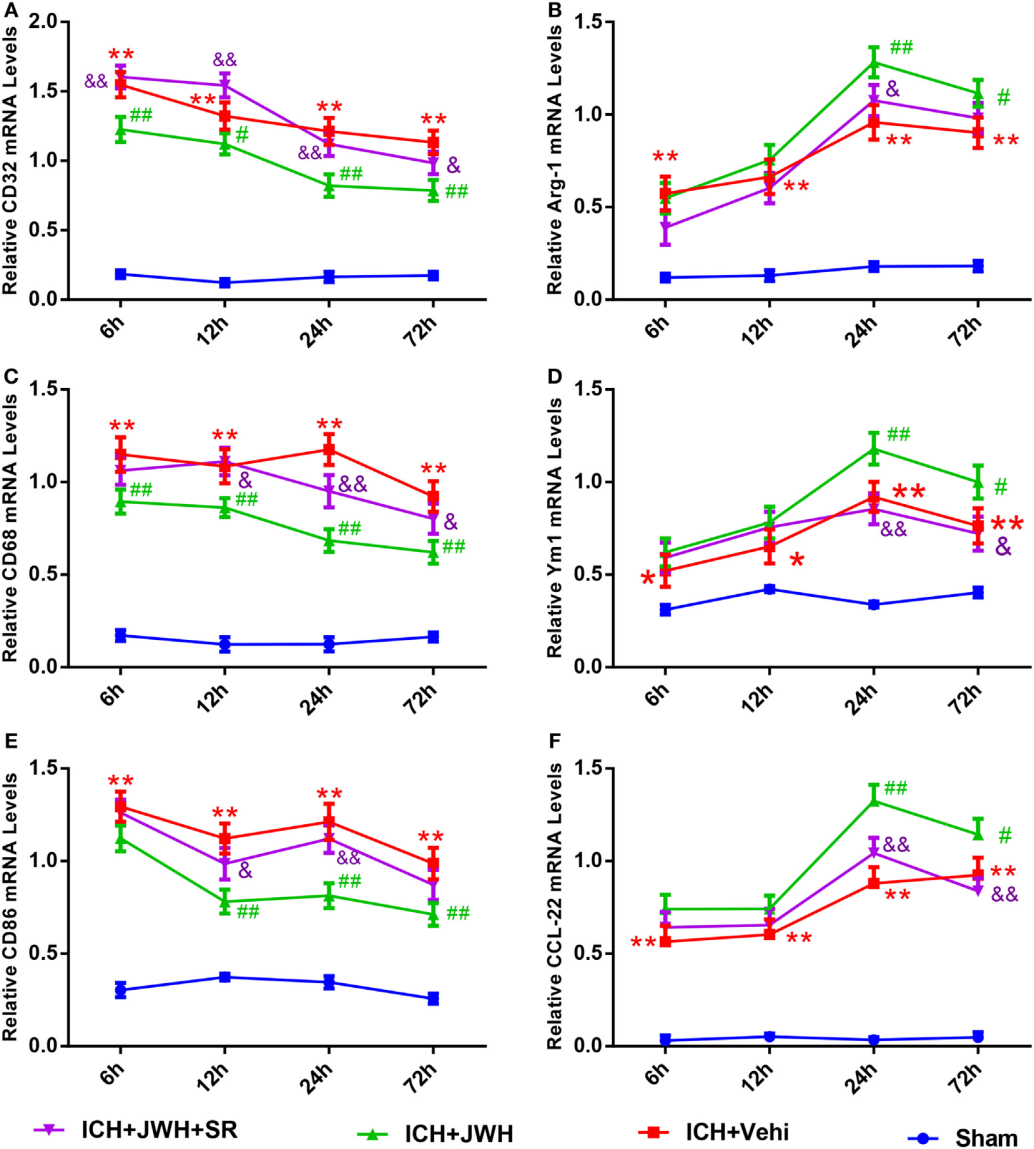
**M1/M2-associated markers of microglia at messenger RNA level**. CD32 **(A)**, CD68 **(C)**, and CD86 **(E)** are M1-associated markers. Arg-1 **(B)**, Ym-1 **(D)**, and CCL-22 **(F)** are M2-associated markers. Values were expressed as mean ± SD, *n* = 6 in each group. *P < 0.05, **P < 0.01 compared with sham group; ^#^*P* < 0.05, ^##^*P* < 0.01 vs. ICH + Vehi group; ^&^*P* < 0.05, ^&&^*P* < 0.01 vs. ICH + JWH group.

**Figure 6:**
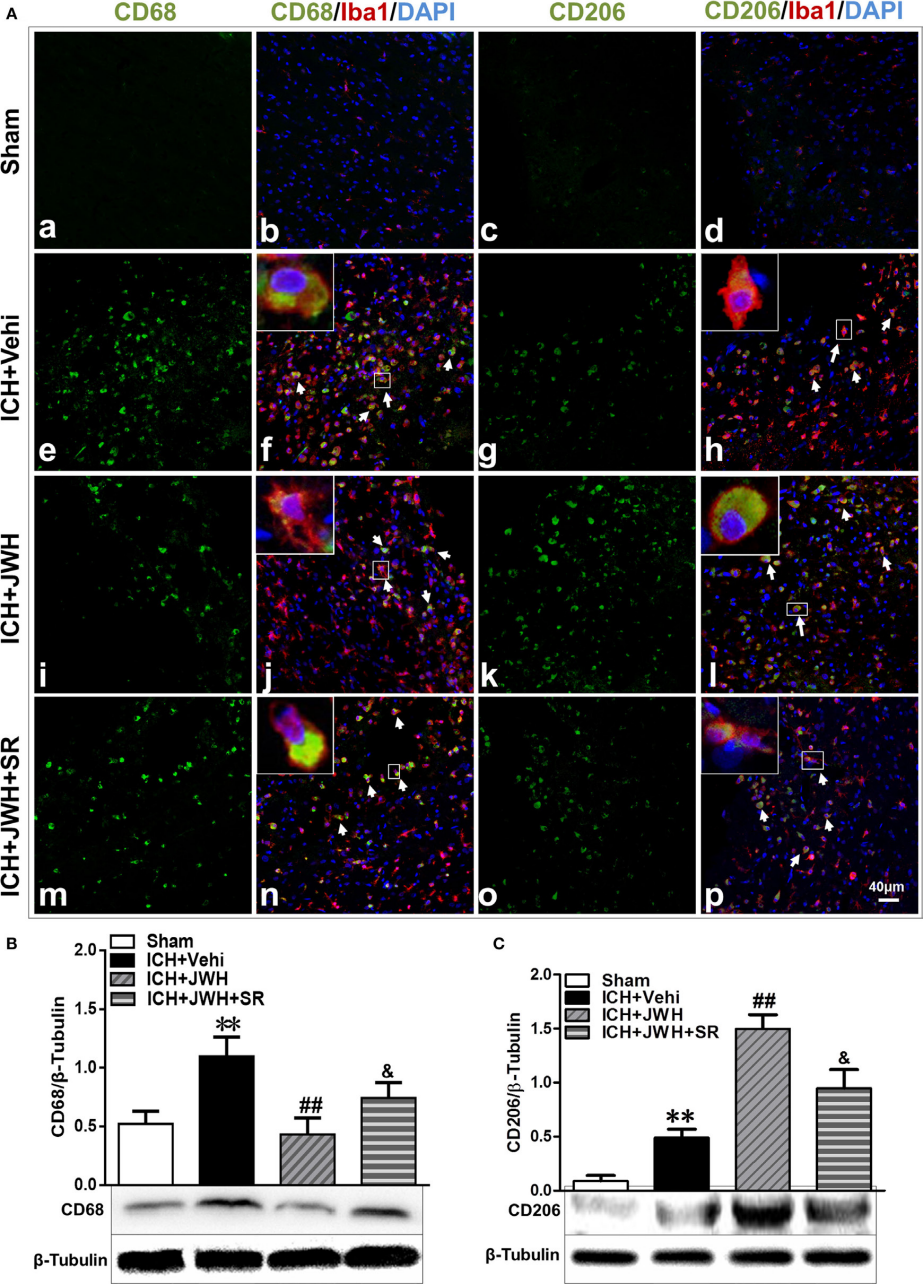
**M1/M2-associated markers of microglia at protein level**. Perihematomal tissue was costained for CD68 (M1 marker) (green) and Iba1 (microglia marker) (red), or CD206 (M2 marker) (green) and Iba1 (red) at 24 h after intracerebral hemorrhage (ICH) **(A)**. Cell nucleus was stained with DAPI (blue). White arrows pointed to typical cells. *n* = 6 in per group. CD68 **(B)** and CD206 **(C)** were also tested using western blots. Values of the relative densitometric analysis were expressed as mean ± SD, *n* = 6 in each group. **P < 0.01 compared with sham group; ^##^*P* < 0.01 compared with ICH + Vehi group; ^&^*P* < 0.05 compared with ICH + JWH group.

To investigate the characteristics of microglial activation during the acute phase following ICH induction, gene levels of M1/M2 markers were investigated time dependently. After ICH, M1-associated markers CD32, CD68, and CD86 increased immediately and peaked at 6 h, and remained a high level to 72 h (Figures 5A,C,E). M2-associated markers Arg-1, Ym-1, and CCL-22 increased slowly and peaked at 24 h, and kept a low level from 6 to 72 h (Figures 5B,D,F). However, the use of JWH133 significantly inhibited the increase of M1-associated mRNA levels (Figures 5A,C,E) and promoted M2-associated mRNA levels (Figures 5B,D,F), with the most obvious differences at 24 h, and all the effects could be significantly reversed by SR144528 (Figures 5A–E). Consistent with the qPCR results, immunofluorescent double-labeled staining and western blot results showed that the M1-associated marker CD68 was also more significantly upregulated than M2-associated marker CD206 at 24 h after ICH (Figures 6A,B). Treatment with JWH133 promoted the protein expression of CD206 and inhibited CD68 expression (Figures 6A–C). These results indicated that CB2R stimulation could drive the acquisition of M2 polarization in microglia and reduce M1 phenotype.

### Inflammatory Responses during ICH Were Inhibited by JWH133 Treatment

**Figure 7:**
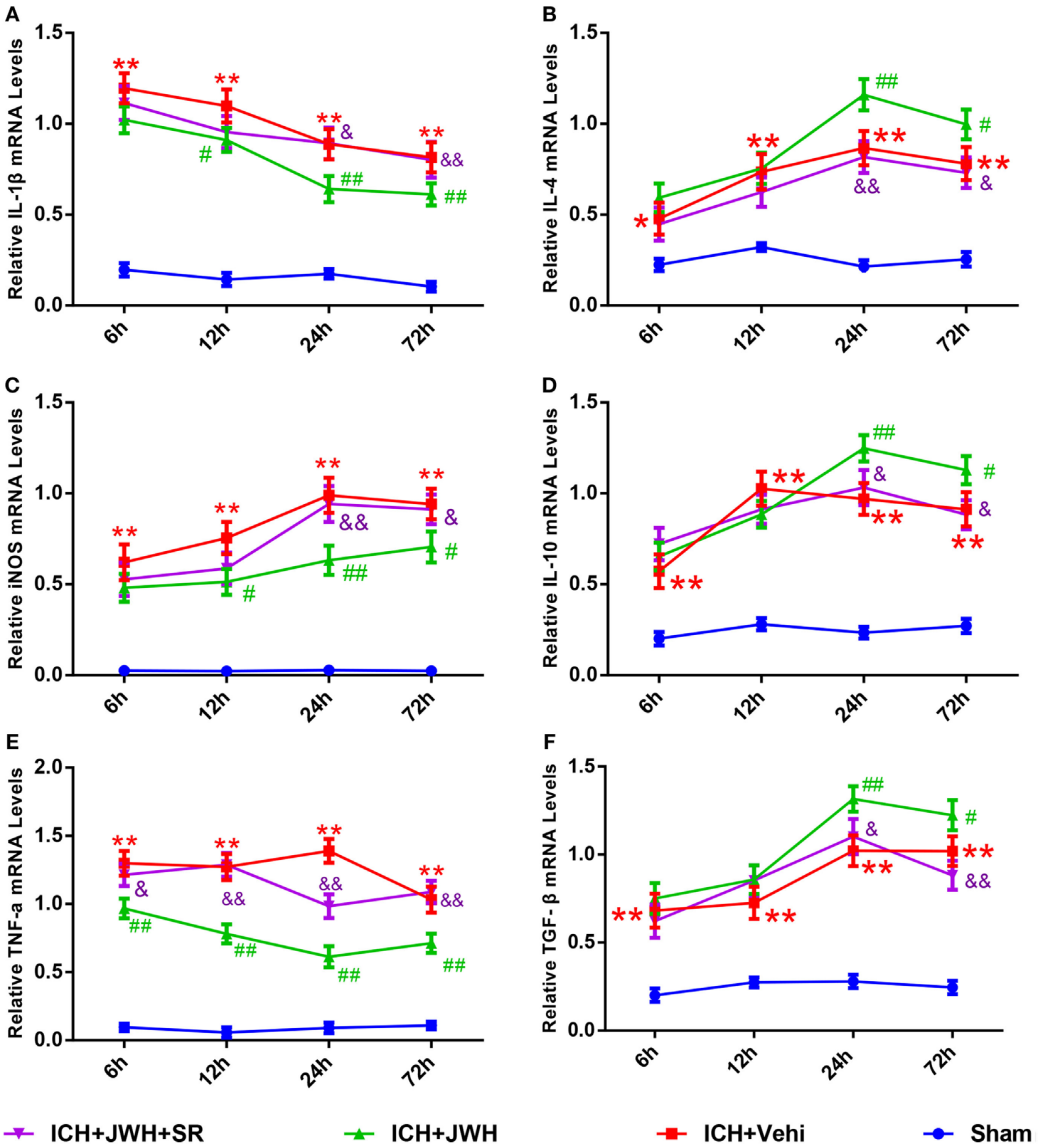
**Inflammation cytokines at messenger RNA level in acute phase after intracerebral hemorrhage (ICH)**. Interleukin-1β **(A)**, iNOS **(C)**, and tumor necrosis factor-α **(E)** are pro-inflammatory cytokines, and the IL-4 **(B)**, interleukin-10 **(D)**, and transforming growth factor beta **(F)** are anti-inflammatory cytokines. The values were mean ± SD, *n* = 6 in each group. *P < 0.05, **P < 0.01 compared with sham group, ^#^*P* < 0.05; ^##^*P* < 0.01 compared with ICH + Vehi group; ^&^*P* < 0.05, ^&&^*P* < 0.01 compared with ICH + JWH group.

Inflammatory cytokine secretion plays an important role in brain injury after ICH. In this study, the mRNA expression levels of both pro-inflammatory (IL-1β, TNF-α, and iNOS) and anti-inflammatory cytokines (IL-4, IL-10, and TGF-β) in perihematomal brain tissue were detected at different time points after ICH. Expression levels of pro-inflammatory cytokines increased immediately and IL-1β peaked at 6 h (Figure 7A), while TNF-α and iNOS (Figures 7C,E) peaked at 24 h post-ICH. The gene levels of IL-4, IL-10, and TGF-β were upregulated at 6 h after ICH and reached a peak at 24 h (Figures 7B,D,F). However, treatment with JWH133 prevented the increase of IL-1β, TNF-α, and iNOS at 6 h after ICH induction, and the effect persisted to 72 h, with the most significant changes occurring at 24 and 72 h (Figures 7A,C,E). In contrast, IL-4, IL-10, and TGF-β were significantly increased at 6 h, with the most significant changes occurring at 24 h (Figures 7B,D,F). All of the JWH133-mediated effects were prevented by treatment with SR144528.

### pPKA–CREB Signaling Pathway Was Upregulated by JWH133 Treatment

**Figure 8:**
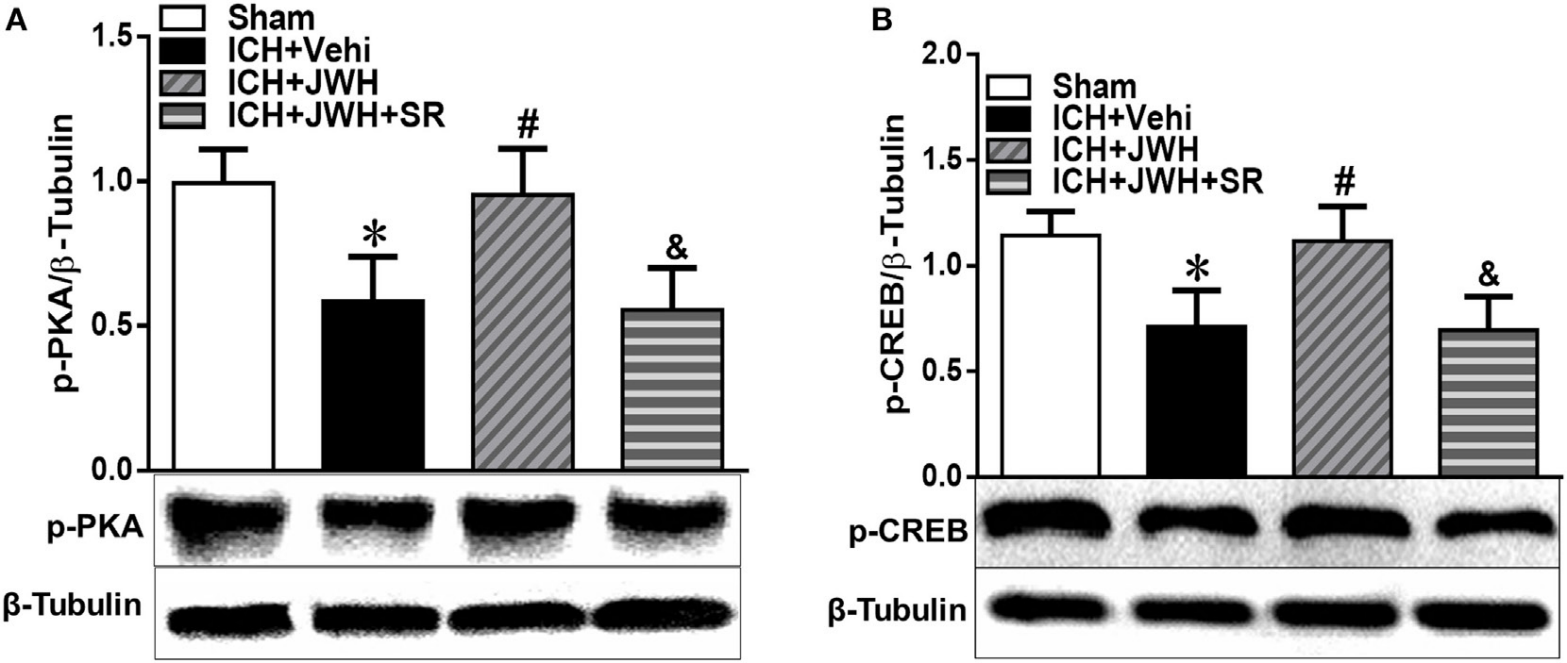
**The expression of pPKA–CREB signaling pathway at 24 h after intracerebral hemorrhage (ICH)**. Total protein around the hematoma was extracted for investigation. Western blot analysis was used to detect the protein expression of **(A)** pPKA and **(B)** pCREB. Values of the relative densitometric analysis were expressed as mean ± SD, *n* = 6. *P < 0.05, **P < 0.01 compared with sham group; ^#^*P* < 0.05, ^##^*P* < 0.01 compared with ICH + Vehi group; ^&^*P* < 0.05, ^&&^*P* < 0.01 compared with ICH + JWH group.

To further explore the underlying mechanisms, we tested the expression of pPKA–CREB signaling pathway. Results of western blot showed that pPKA and pCREB expression was significantly lower in ICH + Vehi group when compared with Sham group (*P* < 0.01), and the ICH + JWH group had significantly higher expression of pPKA (*P* < 0.01) and pCREB (*P* < 0.05) when compared with the ICH + Vehi group (Figures 8A,B), indicating that pPKA–CREB signaling pathway was significantly upregulated by JWH133 treatment. The pretreatment of SR144528 prevented the upregulation effects.

### pPKA–CREB Signaling Pathway Was Involved in the JWH133-Induced Increase of Microglial M2 Polarization

**Figure 9:**
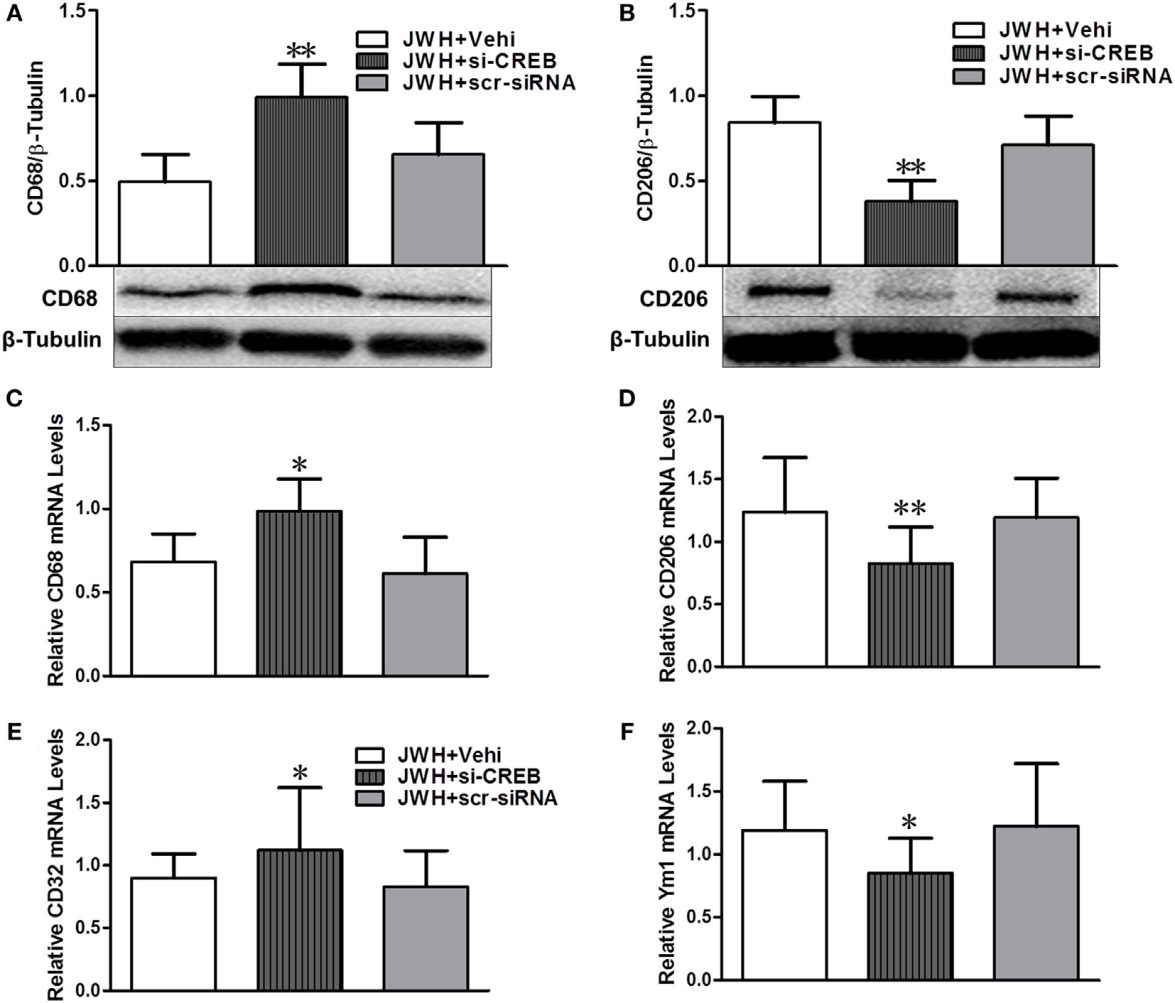
**The relationship between pPKA–CREB signaling pathway and microglial polarization**. Western blot test and relative densitometric analysis (*n* = 6 in each group) of CD68 **(A)** and CD206 **(B)**. Relative messenger RNA level of M1-associated markers CD68 **(C)** and CD32 **(E)**, and M2-associated markers CD206 **(D)** and Ym-1 **(F)** (*n* = 3 in each group). The values were mean ± SD. *P < 0.05, **P < 0.01 compared with JWH + Vehi group.

cAMP-response element binding protein (CREB) *in vivo* knockdown was performed to investigate the potential role of CREB in the effects of JWH133 induced the increase of microglial M2 polarization and the decrease of M1 phenotype. Pretreatment with CREB-1 siRNA sufficiently downregulated the expression of CREB both at gene and protein levels (Figures S1A,B in Supplementary Material). Data showed that CREB-1 siRNA abolished the JWH133-induced increase of M2-associated markers CD206 and Ym-1 at protein or gene level (Figures 9B,D,F), as well as the decrease of M1-associated markers CD68 and CD32 in protein or gene levels (Figures 9A,C,E). These results demonstrated that PKA/CREB signaling pathway plays a crucial role in the JWH133-mediated acquisition of M2 phenotype of microglia.

## Discussion

To our knowledge, this is the first study to demonstrate that JWH133, a selective CB2R agonist, reduces brain water content, neurological deficits, DNA damage, and neuron death in an autologous blood infusion rat ICH model. We also demonstrated that JWH133 suppresses neuroinflammation by driving microglial M2 polarization through PKA/CREB pathway.

Increasing evidence indicates that inflammatory mechanisms are involved in stroke-induced brain injury, and microglia/macrophage activation is thought to play a pivotal pathophysiological role (1, 4). Overactivation of microglia/macrophages can exacerbate the inflammatory response after a stroke, resulting in blood–brain barrier disruption and neuronal damage, but also represents a promising target for stroke treatment. However, broad suppression of microglia/macrophages may deprive the normal physiological defense mechanism of the CNS and lead to unintended side effects. Therefore, an improved understanding of the dual beneficial and detrimental roles of microglia in CNS injury and recovery is critical for improving stroke treatment (11).

Once activated, microglia/macrophages serve as a double-edged sword during the battle between neurological damage and protection. Microglia can be activated to two polarization states: the M1, classically activated phenotype, and the M2, alternatively activated phenotype (28). Upon stimulation with lipopolysaccharide or interferon gamma, microglia can be activated to the M1 phenotype and produce pro-inflammatory mediators, chemokines, redox molecules, costimulatory proteins, and major histocompatibility complex II (29, 30), exacerbating inflammatory damage. Alternatively, microglia can also be activated to the M2 polarization state, which produces anti-inflammatory mediators that promote brain recovery by scavenging cell debris, resolving local inflammation, and are involved in tissue remodeling (31, 32). There is no doubt that it is very important to understand the status and function of polarized microglia activation for assessing inflammation progression and optimizing treatment.

TBI was reported to create a severe cortical lesion, which led to chronic and persistent M1-primed activation that lasted for months to years. On the other hand, ICH often occurs after TBI. However, characteristic of microglial polarization and inflammatory response after ICH remains unclear (33, 34). Xi et al. (11) found that ICH induces microglia activation and polarization in mice. They reported that M1 phenotypic markers were increased and reached a peak as early as 4 h, remained high at 3 days, and decreased 7 days after ICH, whereas M2 phenotypic markers were upregulated later than M1 markers, reaching a peak at day 1 and declined on day 7 after ICH, which is largely consistent with the findings of this study. The results confirmed our hypothesis that in the ICH acute phase, microglia/macrophage were largely activated toward the classical M1 phenotype, and JWH133 influenced the polarization process by promoting the anti-inflammatory M2 phenotype.

We further investigated the intracellular molecular switches that regulate microglia/macrophage polarization. A recent breakthrough in research on microglia/macrophages has revealed that several transcriptional regulators may serve crucial roles in M2-associated marker expression (5, 35). Interferon regulatory factor 4 serves as a key transcriptional factor modulating M2 polarization, whereas the interferon regulatory factor 5 and interferon regulatory factor 8 control macrophages toward M1 polarization. The nuclear hormone receptor peroxisome proliferator-activated receptor γ is an important transcriptional factor that mediates macrophages primed toward M2 polarization (36, 37). CB2R can couple to G_i_ proteins, and CB2R activation has been shown to activate cAMP/PKA (38). Additionally, it has been demonstrated that CB2R stimulation enhances CREB activation after cerebral ischemia through phosphorylation of AMPK (39). JWH133 was also found to attenuate apoptosis by activation of phosphorylated CREB-Bcl-2 pathway after subarachnoid hemorrhage in rats (27). cAMP signaling is spatially and temporally regulated, allowing for the selective activation of a subset of targets. A-kinase anchoring proteins provided the platform for the assembly of signalosomes, which consist of cAMP effectors and their substrates. Among them, the role that cAMP plays in maintaining both microglia and monocyte homeostasis to prevent M1 activation has been reported. PKA was considered to be the cAMP receptor and seem to be closely associated with inflammation (40). Although CREB could be activated by various signaling pathways, including cAMP/PKA, ERK1/2, and PI3K/Akt (41), in this study, we selectively detected PKA/CREB signaling pathway. CB2R agonist JWH133 significantly enhanced the phosphorylated expression of PKA and CREB and knockdown of CREB significantly inversed the JWH133-induced increase of M2 polarization in microglia, indicating that PKA/CREB pathway participates in the effects of CB2R stimulation on microglia polarization during ICH. The results are in agreement with our previous study that the promotion of microglial M2 polarization through the cAMP/PKA pathway participates in the CB2R-mediated anti-inflammatory effects during GMH (26).

Until now, most studies on microglial polarization have focused on TBI and cerebral ischemia, but relatively little was known about the polarization of microglia/macrophages following ICH (31). In this study, we demonstrated the neuroprotective effects of a CB2R agonist via microglial polarization and the underlying molecular mechanisms that involved in microglial polarization, which may represent a new target for ICH therapy.

In summary, our results reveal microglia/macrophage activation and polarization after ICH in rats. Our data showed that the CB2R agonist JWH133 lessened the brain water content, improved neurobehavioral outcomes, and ameliorated DNA damage and neuron death, which resulted in alleviating brain injury after ICH. We also found that JWH133 inhibited the pro-inflammatory cytokine release and promoted microglia M2 polarization, leading to a beneficial anti-inflammatory cytokine release. JWH133 also facilitated the phosphorylation of PKA and its downstream effector, CREB. Moreover, knockdown of CREB *in vivo* abolished the effects of JWH133 on microglial polarization, indicating that the PKA/CREB signaling pathway plays a critical role in the JWH133-mediated effects. Therefore, CB2R may provide a new target to modulate microglia/macrophage-mediated inflammatory injury and recovery after ICH.

## Ethics Statment

All institutional and national guidelines for the care and use of laboratory animals were followed.

## Author Contributions

LL, TY, FZ, NY, TQ, ZH, CQ, TJ, and ZY contribute to the implementation of the experiment. ZG, FH, YY, and CZ contribute to the design and paper writing.

## Conflict of Interest Statement

The authors declare that the research was conducted in the absence of any commercial or financial relationships that could be construed as a potential conflict of interest.
