# Supplementary figures and images for: Inflammatory Regulation by Driving Microglial M2 Polarization: Neuroprotective Effects of Cannabinoid Receptor-2 Activation in Intracerebral Hemorrhage
Source: Front Immunol. 2017 Feb 14;8:112. doi: 10.3389/fimmu.2017.00112 (PMC5306140; doi:10.3389/fimmu.2017.00112)

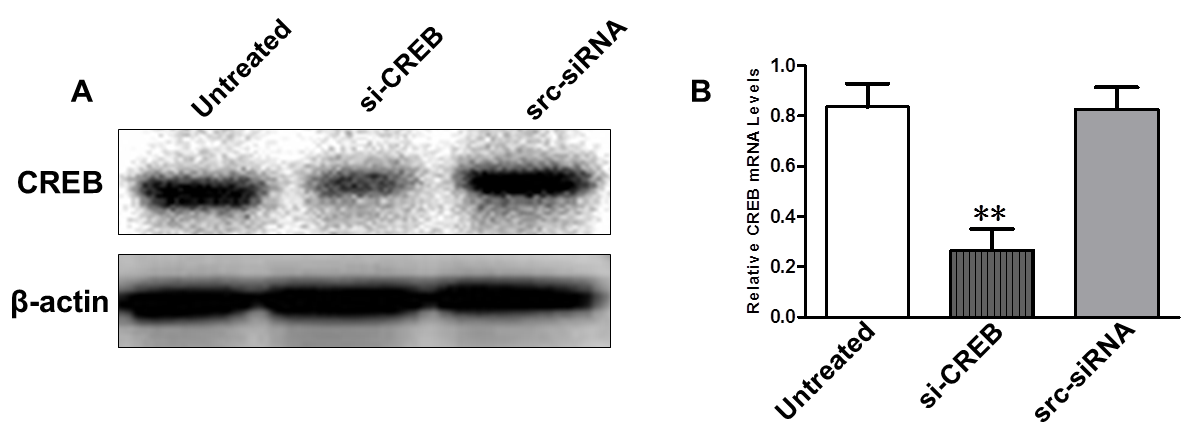

Supplement: Supplementary file 1 [file image_1.tif]
