# Supplementary material for: Inflammatory Regulation by Driving Microglial M2 Polarization: Neuroprotective Effects of Cannabinoid Receptor-2 Activation in Intracerebral Hemorrhage
Source: Front Immunol. 2017 Feb 14;8:112. doi: 10.3389/fimmu.2017.00112 (PMC5306140; doi:10.3389/fimmu.2017.00112)
Supplement: Supplementary file 2 [file presentation_1.pdf]

Inflammatory regulation by driving microglial M2 polarization: neuroprotective effects of cannabinoid receptor-2 activation in intracerebral hemorrhage

Running title: Neuroprotective effects of CB2R in ICH

Li Lin[2†], MD, Tao Yihao[1†], MD, Feng Zhou[1], MD, Niu Yin[1], MD, Tan Qiang[1], MD, Zheng Haowen[4], MD, Chen Qianwei[1], MD, Tang Jun[1], MD, Zhang Yuan[2], MD, Zhu Gang[1], MD, PhD, Feng Hua[1], MD, PhD, Yang Yunfeng[3], MD, PhD, Chen Zhi[1]*, MD, PhD

1. *Department of Neurosurgery, Southwest Hospital, Third Military Medical University, Chongqing, 400038, China*
2. *Department of Neurosurgery, Nanchong Central Hospital, Nanchong 637000, China*
3. *Department of Neurosurgery, Sichuan Provincial Corps Hospital, Chinese People's Armed Police Forces, Leshan, China*
4. *Department of Neurosurgery, Southwest Medical University Affiliated Hospital, Southwest Medical University, Luzhou, 646000, China*

[†]Lin Li and Yihao Tao contributed equally to this work.

*Correspondence:

Zhi Chen

zhichen@tmmu.edu.cn

Figure legend

Figure S1. The expression of CREB at protein and mRNA level at 24 hours after intracerebroventricular injection of CREB-1 siRNA. Tissue was collected from basal ganglia. Western blot was used to assess the protein expression (A) and qPCR was used to test the mRNA level (B). The expression of CREB was significantly down-regulated both at protein and mRNA level. Values of relative mRNA level were expressed as mean ± SD, n = 5 per group. **P < 0.01 compared with Untreated group.
