# Supplementary material for: Inflammatory Regulation by Driving Microglial M2 Polarization: Neuroprotective Effects of Cannabinoid Receptor-2 Activation in Intracerebral Hemorrhage
Source: Front Immunol. 2017 Feb 14;8:112. doi: 10.3389/fimmu.2017.00112 (PMC5306140; doi:10.3389/fimmu.2017.00112)
Supplement: Supplementary file 3 [file table_1.pdf]

Table S1 Forward and reverse sequences of the used primers.

Gene	Forward sequence	Reverse sequence
**CD68**	ATGGTTCCCAGCCATGTGTT	TTTCCACCCTGGGTCAGGTA
**CD86**	GACACCCACGGGATCAATTA	GCCTCCTCTATTTCAGGTTCAC
**CD32**	AATCCTGCCGTTCCTACTGATC	CCTTCGGGCCAAAGATCCTG
**CD206**	ACTGCGTGGTGATGAAAGG	GTGTCACCGTGTCTTCCTTGAG
**Ym1**	GATCACCACCCCTATGACCCT	GGGACCAGTTGGTGTAGTAGC
**Arg-1**	TGGCGTTGACCTTGTCTTGT	TTTGCTGTGATGCCCCAGAT
**CCL-22**	CTGATGCAGGTCCCTATGGT	GCAGGATTTTGAGGTCCAGA
**iNOS**	CAAGCACCTTGGAAGAGGAG	AAGGCCAAACACAGCATACC
**IL-1β**	GGCAACTGTCCCTGAACT	TCCACAGCCACAATGAGT
**TNF-α**	GACCCTCACACTCAGATCATCTTCT	TGCTACGACGTGGGCTACG
**IL-4**	CGTGATGTACCTCCGTGCTT	GTGAGTTCAGACCGCTGACA
**IL-10**	TTGAACCACCCGGCATCTAC	CCAAGGAGTTGCTCCCGTTA
**TGF-β**	TGGCCAGATCCTGTCCAAAC	GTTGTACAAAGCGAGCACCG
