# Supplementary material for: Inflammatory Regulation by Driving Microglial M2 Polarization: Neuroprotective Effects of Cannabinoid Receptor-2 Activation in Intracerebral Hemorrhage
Source: Front Immunol. 2017 Feb 14;8:112. doi: 10.3389/fimmu.2017.00112 (PMC5306140; doi:10.3389/fimmu.2017.00112)
Supplement: Supplementary file 4 [file table_2.pdf]

Table S2 siRNA sequences

	sense	antisence
CREB-1 siRNA	5’-CUGCAGACAUUAACCAUGA [dT][dT]-3’	5′-UCAUGGUUAAUGUCUGCAG [dT][dT]-3′
	5′-CAACCAAGUUGUUGUUCAA[dT][dT]-3′	5′-UUGAACAACAACUUGGUUG [dT][dT]-3′
	5′-GCAAGAGAAUGUCGUAGAA[dT][dT]-3′	5′-UUCUACGACAUUCUCUUGC [dT][dT]-3′
scrambled siRNA	5′-UUCUCCGAACGUGUCACGU[dT][dT]-3′	5′-ACGUGACACGUUCGGAGAA [dT][dT]-3′
